# Follow-up evaluation of the immunological status of children admitted for acute cerebral nervous system infections: a retrospective study

**DOI:** 10.1186/s13052-021-00973-1

**Published:** 2021-02-02

**Authors:** Giulia Spina, Bozzola Elena, Carsetti Rita, Piano Mortari Eva, Cristina Mascolo, Marco Roversi, Villani Alberto

**Affiliations:** 1 University/Hospital Department of Pediatrics, Pediatric and Infectious Diseases Unit, Bambino Gesù Children’s Hospital, IRCCS, Rome, Italy; 2 B cell Physiopathology Unit, Immunology Research Area, Bambino Gesù Children Hospital, Rome, Italy

**Keywords:** Acute cerebral nervous system infections, Children, Immune system

## Abstract

**Background:**

Acute Cerebral Nervous System Infections (ACNS) may cause death or severe complications even to promptly treated children. The role of the immune system in influencing the course and the outcome of meningitis has been studied but it is not yet completely understood. The aim of the research is to ascertain whether children who experienced ACNS infection had a normal immune system.

**Methods:**

Patients under 18 years of age admitted at Bambino Gesù Children from January 2006 till June 2016 for meningitis were asked to participate to the follow-up study. The immune status was evaluated both clinically and by laboratory investigations.

**Results:**

Most of the patients over 3 years at follow up had at least one immunological alteration at follow-up evaluation (74%). Considering ACNS infection etiology, certain pathogens were almost exclusive of patients affected by some immunological alteration, regardless of their age.

**Discussion:**

Our preliminary results indicate that sub-clinical immunological defects may be associated to ACNS pediatric infections. Moreover, to the best of our knowledges, this is the first study correlating pathogens to immune evaluation in ACNS infections. It is, however, important to underline the high frequency of persistent immunological alterations in the analyzed patients. Further studies are needed to confirm our conclusions.

**Conclusions:**

We recommend an immunological assessment at follow up evaluation in children who experienced an ACNS infection.

## Introduction

Acute cerebral nervous system (ACNS) infection are a spectrum of diseases that include cerebellitis, meningitis, encephalitis, and meningoencephalitis.

Bacterial meningitis is a devastating and life-threatening disease, in developing as well as in developed countries. Despite recent advances in antimicrobial therapy and vaccine development, bacterial meningitis still poses a significant cause of morbidity and mortality in populations at risk, such as in infants, children, and the elderly or immunocompromised patients [1, 2]. Although meningitis remains a relatively rare entity in most congenital or acquired immunodeficiencies, the onset of bacterial meningitis and sepsis may be the first and exclusive sign of an underlying immunodeficiency, particularly when other bacteria than *Neisseria meningitidis* (NM) are isolated [3, 4].

The role of the immune system in determining the course and outcome of meningitis is not well understood. Humoral immunodeficiency has been reported in patients with invasive diseases caused by *Streptococcus Pneumoniae* (SP), *Haemophilus Influenzae* (HI) or by other capsulated pathogens [5]. Agammaglobulinemia should also be suspected in a patient with community-acquired bacterial meningitis [4]. Interestingly, Native Americans, Native Alaskans, and African Americans that experience higher rates of invasive bacterial infections due to HI and SP, also show an increased prevalence of congenital immunodeficiencies (such as the Common Variable or Severe Combined Immuno-Deficiency). It is not yet clear whether Toll-Like Receptors 2, 4, and 9 polymorphisms may predict both susceptibility and unfavourable course of bacterial meningitis caused by SP or NM [6–10]. It has, however been demonstrated that TLR- triggered cytokine secretion is involved in the pathogenesis of meningococcal disease [11]. Finally, deficiency of opsonization and phagocytosis, with or without a preserved splenic function, increase the risk of meningitis. Individuals with deficiency of a single complement protein have a greatly increased risk of both carrying the putative pathogen in the nasal mucosa and developing the disease [12]. Altogether, these findings suggest that immune dysregulation and immunodeficiency may predispose to bacterial meningitis.

Few studies conducted on a pediatric population evaluated the risk of developing bacterial meningitis in the presence of any underlying immunodeficiencies.

Aim of the study is to ascertain whether children who experienced ACNS Infections had a normal immune system.

## Materials and methods

### Human subjects

We enrolled patients admitted to the Bambino Gesù Children Hospital, Roma, Italy, between 1st January 2006 and 30th June 2016 with the following diagnosis of ACNS infection: meningitis, meningoencephalitis, encephalitis and cerebellitis. We included all patients younger than 18 years. Exclusion criteria for selection were varicella-zoster virus infection and any of the following comorbidities: neurological disorders, chronic diseases, malignancy, known acquired or congenital immunodeficiency and/or immunosuppressive therapy. All patients underwent at least one follow-up evaluation to check clinical, vaccination and immunological status. To avoid bias due to possible effects of the recent disease, immunological exams were performed at least 1 month after the acute phase. At the follow-up evaluation, all patients were in good clinical condition, without either fever or other acute symptoms.

### Laboratory tests

In details, laboratory tests included measurement of: serum antibodies against vaccine antigens; serum levels of Immunoglobulin (Ig) M, G, and A; complement levels; lymphocyte subpopulations; proliferation and differentiation of B cells. Serum antibodies against vaccine antigens, namely tetanus, HI, SP, *Bordetella pertussis* and *Hepatitis B virus* and serum levels of IgM, IgA and IgG were measured using a commercial ELISA kit for each antigen (Binding Site) and an in-house ELISA respectively. Coating of plates was carried out with an anti-IgM/IgG/IgA (Cat #109-006-064). Peroxidase-conjugated anti-IgM (cat #109-36-129), anti-IgG (cat #109-036-008), or anti-IgA (cat #109-036-011) (all from Jackson Immunoresearch) were used as primary antibodies. Purified serum IgG and IgA, and IgM purified from a myeloma cell line (Jackson Immunoresearch) were used as standards. Peripheral blood mononuclear cells (PBMCs) were isolated from blood on density gradient centrifugation (Lympholyte, CEDARLANE). PBMCs were loaded with CFSE (Life Technologies) to track divided cells. Briefly, 1 × 10^6^ cells /mL were resuspended in PBS 1% FCS and loaded with 1 μM CFSE for 20 min at 37 °C. Cells were then stimulated with 0.25 μM CpG-B ODN2006 (Hycult Biotech). Flow cytometry was then conducted to count the lymphocyte subpopulations and to evaluate proliferation and differentiation of B cells (CD3, CD4, CD8, CD19, CD24, CD38, IgM, CD16/56). Cells were stained with the appropriate combination of fluorochrome-conjugated antibodies, according to standard techniques. Dead cells were excluded from analysis by side/forward scatter gating. At least 50,000 events gated on living cells were analysed, whenever possible, for each sample. Samples were acquired on a BD Fortessa X-20.

The following four criteria were considered diagnostic for immunological alteration: absence of antibodies against vaccine antigens; age-related reduction of immunoglobulin values; reduction of B cell populations, namely memory B cells; defective proliferation and differentiation of B cells.

### Statistics

R, version 3.2.3 (R Foundation for Statistical Computing, Vienna, Austria. http://www.R-project.org/) was used for data analysis. We compared the laboratory results of our patients with age-matched normal values [13, 14]. The t-test was used for comparison of means of Ig and lymphocyte subclass counts. A *p* value less than 0.05 was considered significant.

## Results

In the study period, 127 participants were identified. Of those, we excluded 30 patients because of a concomitant VZV infection. Our final sample included 97 patients affected by ACNS infections. As the efficiency of the immune system varies according to the different age groups, we evaluated the aetiologies and immunological status of ACNS infections in two subpopulations: patients younger than 3 years old (group A) and patients older than 3 years old (group B) at the follow-up evaluation. The demographic and clinical characteristics of our sample are listed in Table 1.

**Table 1:** Characteristics of patients with ACNS infections

Group A		Group B	
Patients (n) 35		Patients (n) 62	
**Gender**		**Gender**	
Male	21 (60%)	Male	38 (61.3%)
Female	14 (40%)	Female	24 (38.7%)
**Age at diagnosis (years)**		**Age at diagnosis (years)**	
Range	0.01–2.43	Range	0.06–17.68
Mean	0.42 (SD 0.62)	Mean	4.07 (SD 4.38)
Age at follow-up (years)		**Age at follow-up (years)**	
Range	0.01–2.43	Range	3.15–19.46
Mean	1.47 (SD 0.81)	Mean	9.82 (SD 4.59)
**Length of hospitalization (days)**		**Length of hospitalization (days)**	
Range	6–492	Range	5–153
Mean	39.26 (SD 83.65)	Mean	30.21 (SD 24.33)
**Type of ACNS infection**		**Type of ACNS infection**	
Meningitis	22 (62.9%)	Meningitis	38 (62.9%)
Meningoencephalitis	7 (20%)	Meningoencephalitis	15 (24.2%)
Encephalitis	5 (14.3%)	Encephalitis	3 (4.8%)
Cerebellitis	1 (2.8%)	Cerebellitis	5 (8.1%)
**Pathogens (n,%)**		**Pathogens (n,%)**	
SP	2 (5.7%)	SP	16 (25.8%)
SA	4 (11.4%)	SA	3 (4.8%)
NM	4 (11.4%)	NM	16 (25.8%)
HI	3 (8.6%)	HI	4 (6.4%)
EC	1 (2.8%)	*E. Coli*	1 (1.6%)
EV	11 (31.4%)	Enterovirus	6 (9.7%)
HHV6	5 (14.3%)	HHV6	3 (4.8%)
EBV	2 (5.7%)	EBV	4 (6.4%)
HSV1	1 (2.8%)	HSV1	1 (1.6%)
TBC	2 (5.7%)	TBC	6 (9.7%)
KP	0 (0%)	*K. Pneumoniae*	1 (1.6%)

*Legend*: *Group A* patients younger than 3 years at follow-up (FUP), *Group B* patients older than 3 years at FUP, *SP* Streptococco Pneumoniae *SA* Streptococco Agalactiae, *NM* Neisseria Meningitidis, *HI Haemophilus Influenzae*, *EC Escherichia Coli*, *EV* Enterovirus, *HHV6* Human Herpes Virus 6, *EBV* Epstein Bar virus, *HSV1* Herpes virus type 1, *TBC Mycobacterium Tuberculosis*, *KP* Klebsiellae Pneumoniae

The majority of our patients had a primary involvement of the meninges, either in the form of meningitis or meningoencephalitis. A similar distribution of the types of infection was found in the two subgroups. The most frequent pathogens isolated in all groups were: NM, SP, *Enterovirus* (EV), *Herpes Human Virus 6* (HHV6) and *Mycobacterium Tuberculosis* (TBC). The relative frequency of the pathogens for each group is shown in Table 1.

Immunoglobulin levels and lymphocytes sub-populations averages were compared with age-related reference values as showed in Tables 2 and 3. At least one immunological alteration as defined by the given criteria was detected in most of our sample, considering patients older than 3 years at the follow-up evaluation (74%). Moreover, 51.4% of the eligible population had some alterations in the B cell proliferation test, while 16 patients had at least one B cell phenotype change (23%). The IgG values were significantly lower than the standard in patients with more than 3 years of age (*p* < 0.05). We did not consider such differences to be significant in patients under 3 years as immunoglobulin levels at this age are greatly variable. As for the lymphocytes sub-populations, CD3 and CD4 counts proved to be significantly lower than the reference values in 11- to 17-years patients (*p* < 0.001); moreover, we found a significantly lower than the reference CD16/56 count in patients of 6 to 10 and 11 to 17 years of age (*p* < 0.01 and *p* < 0.00001 respectively). Routine vaccinations had been administered to most of our population. Nonetheless, we noticed the reduction below the protective values against tetanus, SP, HI and pertussis in 13, 28, 18 and 54% of all cases respectively. Complement levels (C3, C4) and CH50 levels were evaluated in 50 and 20 patients respectively with no statistically significant abnormality revealed.

**Table 2:** Immunoglobulin levels in patients affected by ACNS infection at the follow-up evaluation and comparison with reference values

Age	3–5 years			6–8 years			9–11 years			12–17 years		
		*Ref*	*P-value*		*Ref*	*P-value*		*Ref*	*P-value*		*Ref*	*P-value*
IgA (mg/dL)												
average	119,45	98	NS	118,5	113	NS	155,36	127	NS	146,38	136	NS
SD	49,94			42,58			50,76	14,08		75,16	16,40	
IgG (mg/dL)												
Average	833,18	1117	*P*< 0.01	939,37	1164	*P*< 0.05	1134,64	1164	NS	955,71	1105	*P*< 0.05
SD	146,42			258,93			260,37	72,21		258,54	56,42	
IgM (mg/dL)												
Average	122	119	NS	106,13	121	NS	137,27	129	NS	147,43	132	NS
SD	55,4			41,01			55,61			146,65

**Table 3:** Lymphocytes sub-populations values in patients with ACNS infection older than 3 years of age compared with reference values

Age	3–5 years			6–10 years			11–17 years		
		*Ref*	*P-value*		*Ref*	*P-value*		Ref	*P*-value
CD3 (%)									
medium	67,74	66	NS	68,4	69	NS	69,55	73	*P*< 0.001
SD	4,77			5,51			4,29		
CD4 (%)									
medium	37,71	38	NS	36,85	37	NS	36,83	41	*P*< 0.05
SD	5,85			5,7			6,25		
CD8 (%)									
medium	22,7	23	NS	24,74	25	NS	25,91	26	NS
SD	4,44			5,42			6,76		
CD19 (%)									
medium	20,46	21	NS	16,3	18	NS	14,71	14	NS
SD	6,11			6,25			4,05		
CD16/56 (%)									
medium	10,66	9	NS	13,88	9	*P*< 0.01	14,13	9	*P*< 0.00001
SD	4,18			7,35			4,06		

**Fig. 1:**
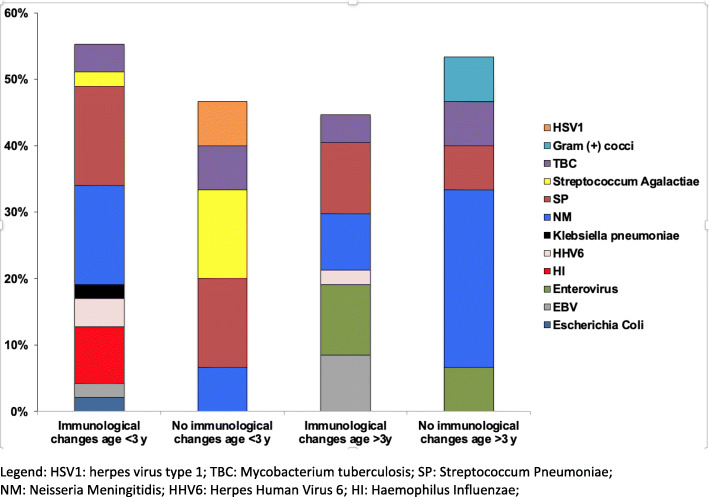
Immunological evaluation and pathogens detected in patients younger than 3 years at follow-up evaluation (Group A) and in patients older than 3 years at follow-up evaluation (Group B). HSV1: herpes virus type 1; TBC: *Mycobacterium tuberculosis; SP: Streptococcum* Pneumoniae; NM: Neisseria Meningitidis; HHV6: Herpes Human Virus 6; HI: *Haemophilus Influenzae*

Considering the etiology of the ACNS infections, we observed that certain pathogens were almost exclusive of patients affected by some immunological alteration, regardless of their age group. *Epstein-Barr Virus* (EBV) and HHV6 were isolated only in patients with at least one immunological alteration (*p* < 0.001). NM has been mostly detected in patients older than 3 years without any immunological alteration. Moreover, 9 different pathogens were observed in patients younger than 3 years old with at least one immunological alteration as showed by Fig. 1. On the other hand, only 5 pathogens were isolated in patients older than 3 years without any immunological changes. The association of a given immunological alteration with the related pathogens are shown in Fig. 1.

## Discussion

In our case series, we found a statistically significant difference in the CD3, CD4 and CD16/56 counts, compared to age-related reference values [14]. In detail, most of the altered values were found in the oldest patients, aged 11–17 years (*p* < 0.05), suggesting that immunity plays an important role in protecting adolescents from ACNS infection. Few studies have highlighted T-lymphocyte alterations in patients with meningitis. In particular, a study conducted on 19 patients documented CD3, CD4 and CD16/56 lower level without any significant differences [15], congruous with other findings [16, 17].

Otherwise, most of the patients had normal complement levels. In literature as well, complements deficiencies are rarely linked to ACNS infections. Complement levels may increase or decrease in ACNS infections depending on the balance between complement production and consumption in each phase of the disease [18].

Immunoglobulin deficiencies are more associated to ACNS infections than primary complement deficiencies [19–21]. Usually, a combined IgG and IgA deficiency or IgG deficiency and neutrophil chemotaxis abnormality have been documented [16] In our study, all patients had normal IgA values while IgM and IgG levels were lower than the age-related standard. More specifically, IgG levels were significantly lower than the reference in most of our sample at the follow-up evaluation (*p* < 0.05).

Considering qualitative immunological tests, we observed that 51.4% of the eligible population had some B-lymphocyte proliferation abnormalities. Other reports have studied functional B cell alterations documenting low levels of B cell proliferations in patients with meningococceal meningitis [22].

To our knowledge, this is the first study correlating the causative pathogens to immune evaluation in ACNS infections. In particular, in our experience, *Escherichia Coli*, *Klebsiella Pneumoniae* and HI were more often isolated in patients younger than 3 years at diagnosis, who had at least one immunological alteration. Moreover, a broader variety of pathogens has been documented in patients with immunological abnormalities at all ages. Typical pathogens, such as NM and SP, were mostly observed in patients without any immune alteration.

Our study has some limitations. As in all retrospective studies, we could not consider the risk of developing the disease in subjects with a given immunodeficiency. Also, we did not have a control group, as we used the normal value reported in literature as a control. Moreover, despite evaluating patients at least 1 month after the admission, while in good clinical conditions, we could not exclude that the immune system dysregulation was a direct consequence of the ACNS infection rather than its cause. Finally, our study was based on a relatively small sample size, owing to the rarity of the disease.

Further studies are necessary to confirm our results as to evaluate the causal relationship between immunological status and clinical sequelae in patients with ACNS infections.

## Conclusion

We suggest that an immunological evaluation should be performed in pediatric patients with ACNS infections. In fact, our preliminary results indicate that ACNS infections occur in children who have subclinical, but measurable immunological alterations.

More specifically, we suggest a quantitative assessment of B cells, IgA, IgM and IgG in patients with a previous bacterial meningitis, at least 1 month after the onset of the disease or later, to avoid bias due to possible effects of the recent disease. Moreover, complement evaluation should also be considered in patients who experienced NM infection or recurrent infections.

Finally, atypical pathogens should be searched in patients with a suspected immunological alteration.

## Data Availability

At Bambino Gesù Children Hospital.

## Abbreviations

ACNS: Acute Cerebral Nervous System Infections (ACNS)
NM: Neisseria meningitidis
SP: *Streptococcus Pneumoniae*
HI: *Haemophilus Influenzae*
Ig: Immunoglobulin
EBV: Epstein-Barr Virus
EV: Enterovirus
HHV6: Herpes Human Virus 6
TBC: Mycobacterium Tubercoulosis
