# Correction to: Follow-up evaluation of the immunological status of children admitted for acute cerebral nervous system infections: a retrospective study.

**DOI:** 10.1186/s13052-021-01171-9

**Published:** 2021-10-29

**Authors:** Giulia Spina, Elena Bozzola, Rita Carsetti, Eva Piano Mortari, Cristina Mascolo, Marco Roversi, Alberto Villani

**Affiliations:** 1 University/Hospital Department of Pediatrics, Pediatric and Infectious Diseases Unit, Bambino Gesù Children’s Hospital, IRCCS, Rome, Italy; 2 B cell Physiopathology Unit, Immunology Research Area,Bambino Gesù Children Hospital, Rome, Italy

Following the publication of the original article [1], the authors identified an error in the author names of Elena Bozzola, Rita Carsetti, Eva Piano Mortari, and Alberto Villani. The given name and family name were erroneously transposed.

The incorrect author names are: Bozzola Elena, Carsetti Rita, Piano Mortari Eva and Villani Alberto.

The correct author names are: Elena Bozzola, Rita Carsetti, Eva Piano Mortari, and Alberto Villani.

The author group has been updated above and the original article [1] has been corrected.
